# Impact of Diet and Drugs on Fecal *Lachnoclostridium* Gene Marker (*m3*) in Non‐Invasive Diagnosis of Colorectal Neoplasia

**DOI:** 10.1111/jgh.70295

**Published:** 2026-02-13

**Authors:** Min Dai, Louis H. S. Lau, Haiyun Shi, Yang Sun, Xiaobo Li, Mengbin Li, Hui Wang, Raphaela I. Lau, Jessie Q. Y. Liang, Francis K. L. Chan, Siew C. Ng

**Affiliations:** ^1^ Microbiota I‐Center (MagIC) Shatin Hong Kong SAR China; ^2^ Department of Medicine and Therapeutics The Chinese University of Hong Kong Shatin Hong Kong SAR China; ^3^ Li Ka Shing Institute of Health Sciences, State Key Laboratory of Digestive Disease, Institute of Digestive Disease The Chinese University of Hong Kong Shatin Hong Kong SAR China; ^4^ Department of Gastroenterology, Beijing Friendship Hospital Capital Medical University, National Clinical Research Center for Digestive Disease, Beijing Digestive Disease Center, State Key Laboratory of Digestive Health Beijing China; ^5^ Department of Gastroenterology The First Affiliated Hospital of Kunming Medical University, Yunnan Province Clinical Research Center for Digestive Disease, Yunnan Geriatric Medical Center Kunming China; ^6^ Division of Gastroenterology and Hepatology, Shanghai Institute of Digestive Disease, NHC Key Laboratory of Digestive Diseases, Renji Hospital Shanghai Jiaotong University School of Medicine Shanghai China; ^7^ Department of Digestive Surgery, Xijing Hospital of Digestive Diseases Air Force Medical University Xi'an China; ^8^ Guangdong Institute of Gastroenterology, Guangdong Provincial Key Laboratory of Colorectal and Pelvic Floor Diseases The Sixth Affiliated Hospital, Sun Yat‐sen University Guangzhou China; ^9^ Key Laboratory of Human Microbiome and Chronic Diseases (Sun Yat‐sen University), Ministry of Education Guangzhou China; ^10^ Centre for Gut Microbiota Research The Chinese University of Hong Kong Shatin Hong Kong SAR China

**Keywords:** biomarker, colorectal neoplasia, diet, drug, *Lachnoclostridium*

## Abstract

**Background:**

A novel bacterial gene marker, *Lachnoclostridium* (*m3*), has potential for non‐invasive diagnosis of colorectal cancer (CRC) and adenoma.

**Aim:**

This study aims to determine the diagnostic performance of *m3* in a multi‐center cohort and to examine the effect of diet and drugs on fecal *m3* in subjects with colorectal neoplasia.

**Methods:**

In a prospective cohort study, we included 2563 subjects (330 CRC, 1119 adenomas, and 1114 controls) from two independent cohorts from Hong Kong and five cities in the Chinese mainland. Stool samples were collected before colonoscopies and bowel preparation. Dietary information was collected using a qualitative dietary questionnaire. Fecal *m3* was measured using quantitative PCR (qPCR). Receiver operating characteristic (ROC) curves were used to evaluate the diagnostic performance of fecal *m3*. The impact of diet and drugs on fecal *m3* was investigated using in vitro experiments and multivariate analysis by logistic regression.

**Results:**

Fecal *m3* could significantly discriminate CRC and advanced adenoma (AA) from normal controls, with areas under the receiver operating characteristic curve (AUROC) of 0.701 and 0.604 (both *p* < 0.001), respectively. Three types of dietary components (meat, vegetables, and oil) and 15 drugs did not affect fecal *m3* levels in vitro. In multivariate analysis, after adjusting for clinical factors, diet and drugs did not have a significant impact on fecal *m3* levels in subjects with colorectal neoplasia.

**Conclusion:**

Fecal *m3* levels and diagnostic performance were not influenced by diet and drugs. It can be considered a promising and reliable non‐invasive diagnostic biomarker for colorectal neoplasia.

## 1 Introduction

Colorectal cancer (CRC) is prevalent globally [1], and studies have shown that the composition and diversity of the gut microbiome differ significantly between healthy individuals and those with CRC [2, 3, 4]. This dysbiosis can be a key factor in CRC development and progression [5, 6]. Metagenomic analysis of fecal samples demonstrated that specific bacterial species (e.g., *Fusobacterium nucleatum* , *Peptostreptococcus stomatis* ) [7] and certain bacterial gene markers such as *F. nucleatum* (*Fn*), *Clostridium hathewayi* (*Ch*), and *Bacteroides clarus* (*Bc*) have potential as non‐invasive biomarkers for early CRC diagnosis [8]. Advantages of stool markers are that they are less invasive than traditional methods like colonoscopy, with comparable performance to allow for early detection and intervention. For example, a novel fecal bacterial gene marker labeled as *m3* from the *Lachnoclostridium* family has been reported to have the capacity to detect early CRC [9]. Fecal levels of *m3* showed a significant linear trend, increasing from controls to adenoma to CRC [9]. The combination of *m3* with the other three known CRC‐related biomarkers (*Fn*, *Ch*, and *Bc*) showed good performance in diagnosing CRC, with an area under the receiver operating characteristic (AUROC) curve of 0.91 [9]. The combined score of the four biomarkers also showed superior sensitivity for CRC and advanced adenoma (AA) detection than fecal immunochemical test (FIT) in the asymptomatic cohort [10]. Importantly, *m3* alone performed better in diagnosing adenomas than other CRC‐related biomarkers (*Fn* and *Ch*) [9]. However, diet, including common types of foods consumed and dietary patterns, may rapidly alter the composition and function of gut microbiota [11]. Similarly, drugs, such as antibiotics and other medications for chronic diseases such as diabetes, can directly or indirectly influence the diversity and abundance of microorganisms in the gut [12, 13]. These potential confounding factors may affect the levels of microbiome markers and diagnostic performance; therefore, they have to be investigated and validated before being considered as reliable biomarkers.

In this study, we aimed to determine the diagnostic performance of a novel fecal bacterial gene marker, *m3*, and to assess the impact of diet and drugs on fecal *m3* for colorectal neoplasia detection, using a multi‐center prospective cohort.

## 2 Methods

### 2.1 Study Design

This study consisted of two independent prospective cohorts from different geographical locations. Cohort I included 1765 subjects (45 CRC, 919 adenomas, and 801 normal controls) recruited at the Prince of Wales Hospital in Hong Kong from June 2022 to September 2024. Cohort II included 798 subjects (285 CRC, 200 adenomas and 313 normal controls) recruited from five hospitals (including Beijing Friendship Hospital affiliated to Capital Medical University, the First Affiliated Hospital of Kunming Medical University, the Sixth Affiliated Hospital of Sun Yat‐sen University, Renji Hospital affiliated to Shanghai Jiaotong University School of Medicine, and Xijing Hospital) from July 2022 to December 2023 in the Chinese mainland. This study was approved by the Joint Chinese University of Hong Kong–New Territories East Cluster Clinical Research Ethics Committee (CREC reference no. 2022.170) and the Ethics Committee of Beijing Friendship Hospital, Capital Medical University (No. 2022‐P2‐084).

### 2.2 Subjects

All subjects were aged ≥ 18 years and required elective colonoscopy for CRC screening, or polyp surveillance, investigation of symptoms (e.g., anemia, change in bowel habit, abdominal pain), or endoscopic resection (e.g., endoscopic mucosal resection or endoscopic submucosal dissection) for AA or early‐stage CRC. Written informed consent was obtained from all subjects. Subjects were excluded if they had contraindications to colonoscopy (e.g., perforation, intestinal obstruction, unstable cardiopulmonary status), contraindications to polyp resection (e.g., active gastrointestinal bleeding, uninterrupted anticoagulation or dual antiplatelets), previous colonic resection, personal history of CRC, polyposis syndrome, or inflammatory bowel disease, or advanced comorbid conditions (defined as American Society of Anesthesiologists grade 4 or above).

### 2.3 Sample Collection and Endoscopic Diagnosis

Subjects were instructed to collect stool samples in a standardized stool collection tube with preservative at home. Stool collection tubes were returned within 1 month to our laboratory before the bowel preparation and scheduled colonoscopy. Antibiotics or probiotics were prohibited within 30 days before sample collection. The final diagnosis was based on colonoscopy examination. Histopathological review was conducted by independent pathologists for biopsies obtained or lesions removed. Subjects were categorized as normal (control group) if the colonoscopy showed no lesions or only non‐neoplastic findings. They were classified into the adenoma group (if histology confirmed adenoma with dysplasia) and the CRC group (if histology confirmed adenocarcinoma). AA was defined as an adenoma ≥ 10 mm, and/or with a villous component ≥ 20%, and/or harboring high‐grade dysplasia.

### 2.4 Clinical and Dietary Information Collection

In Cohort I, baseline demographics and clinical information, including age, gender, body height and weight, smoking and alcohol status, medical history and comorbidities, drug intake, and family history, were collected by a questionnaire and verified by hospital records. Dietary information was collected using a self‐administered qualitative food intake questionnaire (Data S1). This questionnaire assessed subjects' dietary habits over the past 2 weeks. It included 13 categories of food and drinks (e.g., meats, vegetables, baked foods, milk and dairy products, alcoholic drinks, etc.) in which respondents indicated whether they consumed these items.

### 2.5 Stool DNA Extraction and Quantitation of Fecal *m3* Levels by qPCR

For samples in Cohort I, stool DNA extraction was performed using the Norgen Stool DNA Isolation Kit (Norgen Biotek Corp), following the manufacturer's instructions. For samples in Cohort II, stool DNA extraction was performed using the BayBiopure Magnetic Stool Nucleic Acid Kit (Baybio, STNM‐IVD‐48‐K or Baybio, NE‐02‐K‐96) following the manufacturer's instructions. Cross‐validation using different DNA extraction kits by our team found no difference in qPCR results for the same sample (data on file). The design of primer and probe sequences was described in our previous studies [9, 14]. For samples in Cohort I, qPCR amplifications were performed on an ABI QuantStudio sequence detection system with thermal cycler parameters of 95°C for 10 min and (95°C for 15 s, 60°C for 1 min) × 45 cycles and for samples in Cohort II with thermal cycler parameters of 95°C for 30 s and (95°C for 15 s, 60°C for 1 min) × 40 cycles. The effect of different qPCR cycle numbers for the bacterial gene marker *m3* was assessed, and the results indicated that cycle numbers between 40 and 45 did not affect the qPCR results (Data S2). Positive controls for the markers and a negative control (H_2_O as template) were included in every experiment. The relative level of *m3* was calculated using the delta Cq method as compared to the internal control with ΔCq = Cqtarget − Cqcontrol. Relative abundances = POWER (2, −ΔCq). To allow for easier comparison and visualization of the results, particularly when dealing with low abundance measurements, the results were shown as the log value of “*10e6 + 1” following our previous report [9, 10].

### 2.6 In Vitro Experiments to Evaluate the Effects of Diet and Drugs on Fecal *m3* Levels

We selected three stool samples from Cohort II that tested negative and with Ct levels at 30–32 (defined as *m3*‐moderate‐positive) and at 33–35 (defined as *m3*‐weak‐positive) for fecal *m3* and performed Sanger sequencing on the three samples to ensure the positive and negative results of Sanger sequencing were consistent with those of qPCR. We aliquoted and thoroughly mixed the stool samples with the three types of targeted dietary components (meat, vegetables, and oil) and 15 types of drugs for upper respiratory tract infection and gastrointestinal symptoms, respectively. These drugs included vitamins, domperidone, ranitidine, cimetidine, berberine, glycerin, paracetamol, ibuprofen, banlangen, omeprazole, and indomethacin. The concentration of each diet and drug was listed in Table S1. Each aliquoted stool sample, without being treated with diets or drugs, was set up as a negative control. DNA extraction and qPCR test were performed to test the fecal *m3* levels. Each aliquoted sample mixed with diets or drugs was tested in triplicate, and |ΔCt| = Cttreated − Ctcontrol was used to describe the difference in fecal *m3* level between diet‐ or drug‐treated samples and controls. We performed a pilot experiment to determine the range |ΔCt| threshold and set ΔCt| < 1.0 and |ΔCt| < 1.5 as “no effect” for *m3*‐moderate‐positive and *m3*‐weak‐positive samples, respectively (Data S2).

### 2.7 Statistics

Data were analyzed using R software (4.3.3; R Foundation for Statistical Computing, Vienna, Austria) or MedCalc Statistical Software V.23.4 (MedCalc Software bvba, Ostend, Belgium). Continuous variables were presented as mean (standard deviation, SD), whereas categorical variables were shown as number (percentage). The prevalence of fecal *m3* among different groups was compared using Chi‐square test or Fisher's exact test (expected count < 5). The relative levels of fecal *m3* among different groups were compared using Wilcoxon rank‐sum test (Mann–Whitney *U* test). ROC curves were used to evaluate the diagnostic value of fecal *m3* in distinguishing CRC/AA and normal controls. The best cut‐off values were determined by ROC analyses that maximized the Youden index (J = Sensitivity + Specificity − 1), taking into account the clinical requirements for both detection sensitivity and specificity [15]. Logistic regression was used to conduct univariate and multivariate analysis for the association between the relative level of fecal *m3* and clinical, drug or diet factors. A two‐sided *p*‐value of less than 0.05 was regarded as statistically significant.

## 3 Results

### 3.1 Characteristics and Prevalence of Fecal *m3* in Colorectal Neoplasia

**FIGURE 1:**
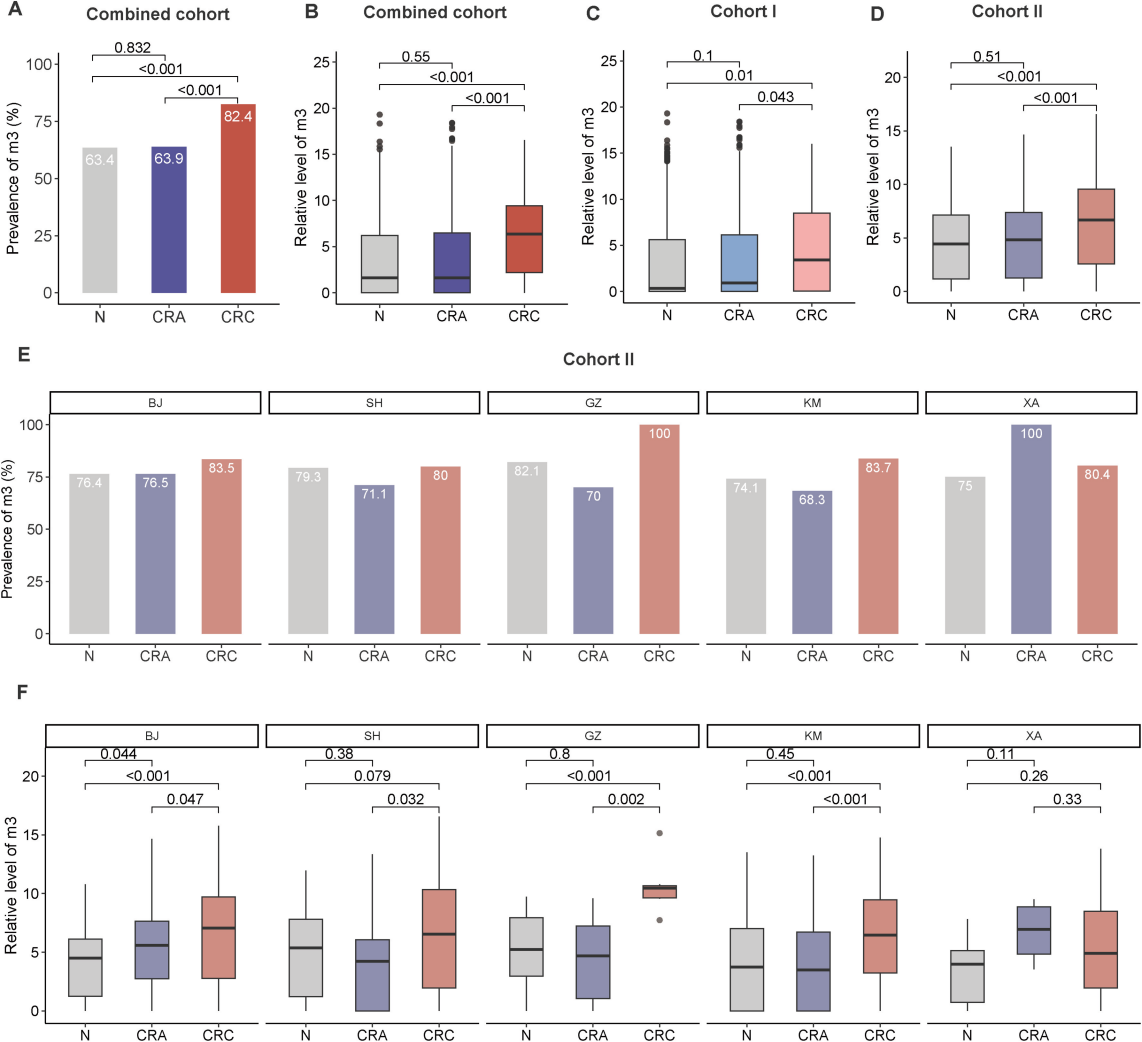
Prevalences (A) and relative levels (B) of fecal *m3* in the combined cohort (Cohorts I and II) among normal controls (N), colorectal adenoma (CRA), and colorectal cancer (CRC) groups. Relative levels of fecal *m3* in Cohort I (C) and Cohort II (D) among normal controls, CRA, and CRC groups. Prevalences (E) and relative levels (F) of fecal *m3* among normal controls, CRA, and CRC groups in five cities (Beijing, BJ; Shanghai, SH; Guangzhou GZ; Kunming, KM; and Xi'an, XA) of the Chinese mainland.

A total of 1765 subjects (45 CRC, 919 adenomas and 801 normal controls) were included in Cohort I (Table 1). The mean age of subjects with CRC, adenoma, and normal controls in Cohort I was 70.4 ± 8.0, 68.4 ± 7.4, and 65.5 ± 9.6 years, respectively. A total of 798 subjects (285 CRC, 200 adenomas, and 313 normal controls) were included in Cohort II (Table S2). The mean age of subjects with CRC, adenoma, and normal controls in Cohort II was 61.2 ± 8.3, 59.1 ± 9.1, and 55.0 ± 8.9 years, respectively. When we combined Cohorts I and II, the prevalence of fecal *m3* was 82.4%, 63.9%, and 63.4% in CRC, adenoma, and normal controls, respectively (Figure 1A). Both the prevalence and relative levels of fecal *m3* were significantly higher in CRC than in adenoma (*p* < 0.001) or normal controls (*p* < 0.001) (Figure 1A,B). Both the prevalence and relative levels of fecal *m3* in AA were significantly higher than in non‐AA (prevalence, *p* = 0.001; relative level, *p* < 0.001) or normal controls (prevalence, *p* < 0.001; relative level, *p* < 0.001) (Figure S1A,B). The same trend was also observed in the relative levels of fecal *m3* in Cohort I (Figure S1C) and Cohort II (Figure S1D), respectively. The prevalence of fecal *m3* was 88.5%, 81.4%, 82.1%, and 87.5% in CRC stages I–IV, respectively (Figure S1E). There was no significant difference in the prevalence or relative level of fecal *m3* among different CRC stages (Figure S1E,F). In Cohort II, the prevalences of fecal *m3* among CRC, adenomas, and normal controls across five cities (Beijing, Shanghai, Guangzhou, Kunming, and Xi'an) in the Chinese mainland were 80%–100%, 68.3%–100%, and 74.1%–82.1%, respectively (Figure 1E). The relative levels of fecal *m3* were consistently higher in CRC compared with adenomas in the four cities (Beijing, Shanghai, Guangzhou, and Kunming) in the Chinese mainland, and they were significantly higher in CRC compared with normal controls in three cities (Beijing, Guangzhou, and Kunming) in the Chinese mainland. Significant differences in the relative level of fecal *m3* between adenoma and normal controls were only observed in Beijing samples (Figure 1F).

**TABLE 1:** Baseline characteristics of Cohort I.

Variable	All (*N* = 1765)	Normal (*N* = 801)	Adenoma (*N* = 919)	CRC (*N* = 45)
Age, mean (SD), year	67.1 (8.6)	65.5 (9.6)	68.4 (7.4)	70.4 (8.0)
Gender, male (%)	881 (49.9)	326 (40.7)	529 (57.6)	26 (57.8)
BMI, mean (SD)	23.0 (3.9)	23.5 (3.8)	24.3 (4.0)	23.6 (3.8)
Smoking history, (%)				
Current smoker	144 (8.2)	46 (5.7)	96 (10.4)	2 (4.4)
Ex‐smoker	297 (16.8)	113 (14.1)	171 (18.6)	13 (28.9)
Never	1324 (75.0)	642 (80.1)	652 (70.9)	30 (66.7)
Drinking history, *n* (%)				
Current drinker	182 (10.3)	73 (9.1)	103 (11.2)	6 (13.3)
Ex‐drinker	105 (5.9)	38 (4.7)	65 (7.1)	2 (4.4)
Never	1478 (83.7)	690 (86.1)	751 (81.7)	37 (82.2)
Comorbidities, *n* (%)				
Hypertension	994 (56.3)	394 (49.2)	570 (62.0)	30 (66.7)
Diabetes mellitus	508 (28.8)	174 (21.7)	317 (34.5)	17 (37.8)
Hyperlipidemia	656 (37.2)	275 (34.3)	364 (39.6)	17 (37.8)
Family history of CRC, *n* (%)	237 (13.4)	99 (12.4)	133 (14.5)	5 (11.1)
Concurrent drugs, *n* (%)				
Statins	772 (43.7)	293 (36.6)	460 (50.1)	19 (42.2)
Metformin	291 (16.5)	99 (12.4)	187 (20.3)	5 (11.1)
Aspirin	251 (14.2)	99 (12.4)	146 (15.9)	6 (13.3)

Abbreviations: BMI, body mass index; CRC, colorectal cancer; SD, standard deviation.

### 3.2 Diagnostic Performance of Fecal *m3* in Advanced Colorectal Neoplasia

**FIGURE 2:**
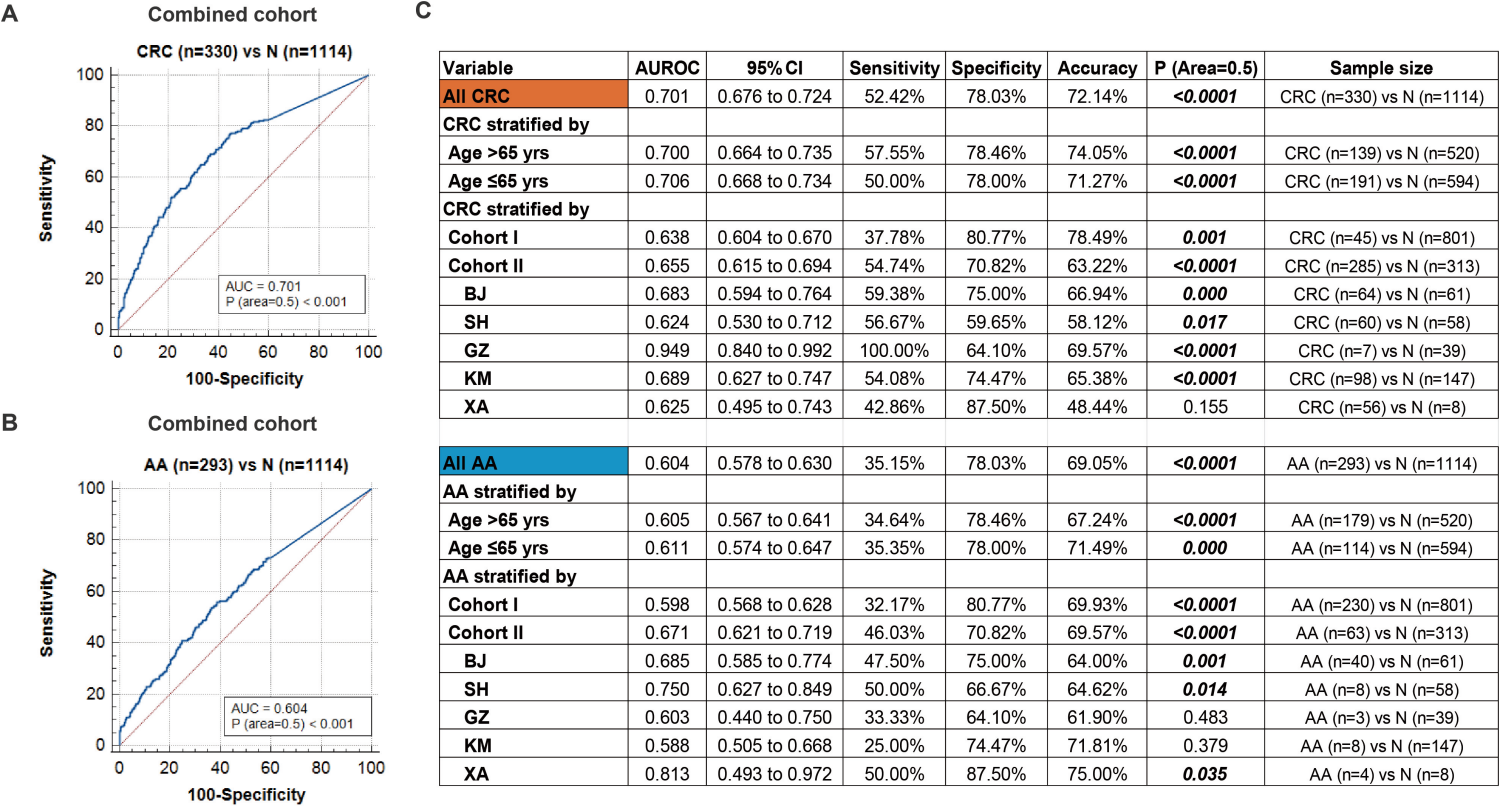
Receiver operating characteristic (ROC) curves of fecal *m3* in discriminating patients with CRC (A) and AA (B) from normal controls in the combined cohort. (C) Diagnostic values of fecal *m3* in discriminating patients with CRC and AA from normal controls with stratification by age > 65 and ≤ 65 years old, Cohort I and Cohort II, and different cities in Cohort II. AA, advanced adenoma; AUROC, area under the receiver operating characteristic curve; BJ, Beijing; CI, confidence interval; CRC, colorectal cancer; GZ, Guangzhou; KM, Kunming; N, normal control; SH, Shanghai; XA, Xi'an.

As AA is considered a clinically important phase in the prevention of CRC, we assessed the diagnostic performance of fecal *m3* in detecting AA and CRC. ROC curve analysis showed that fecal *m3* could significantly discriminate CRC and AA patients from normal controls in the combined cohort, with AUROC of 0.701 and 0.604 (both *p* < 0.001), respectively (Figure 2A,B). The performance of fecal *m3* was consistent across age groups for both CRC and AA, with AUROC values of 0.700 and 0.605 for patients over 65 years (both *p* < 0.001) and AUROC values of 0.706 and 0.611 for those 65 years or younger (both *p* < 0.001) (Figure 2C). Fecal *m3* showed similar performance for CRC among Cohorts I and II, with AUROC values of 0.638 (*p* = 0.001) in Cohort I and 0.655 (*p* < 0.001) in Cohort II, respectively (Figure 2C), and exhibited a numerically better AUROC for AA in Cohort II (0.671, *p* < 0.001) compared to Cohort I (0.598, *p* < 0.001) (Figure 2C). Across different cities in Cohort II, the AUROCs of fecal *m3* for CRC and AA were 0.625–0.949 and 0.588–0.813, respectively (Figure 2C). In the combined cohort, at a specificity of 78.03%, the sensitivities of fecal *m3* for CRC and AA were 52.42% and 35.15%, respectively (Figure 2C).

### 3.3 Impact of Diet on Fecal *m3*

**FIGURE 3:**
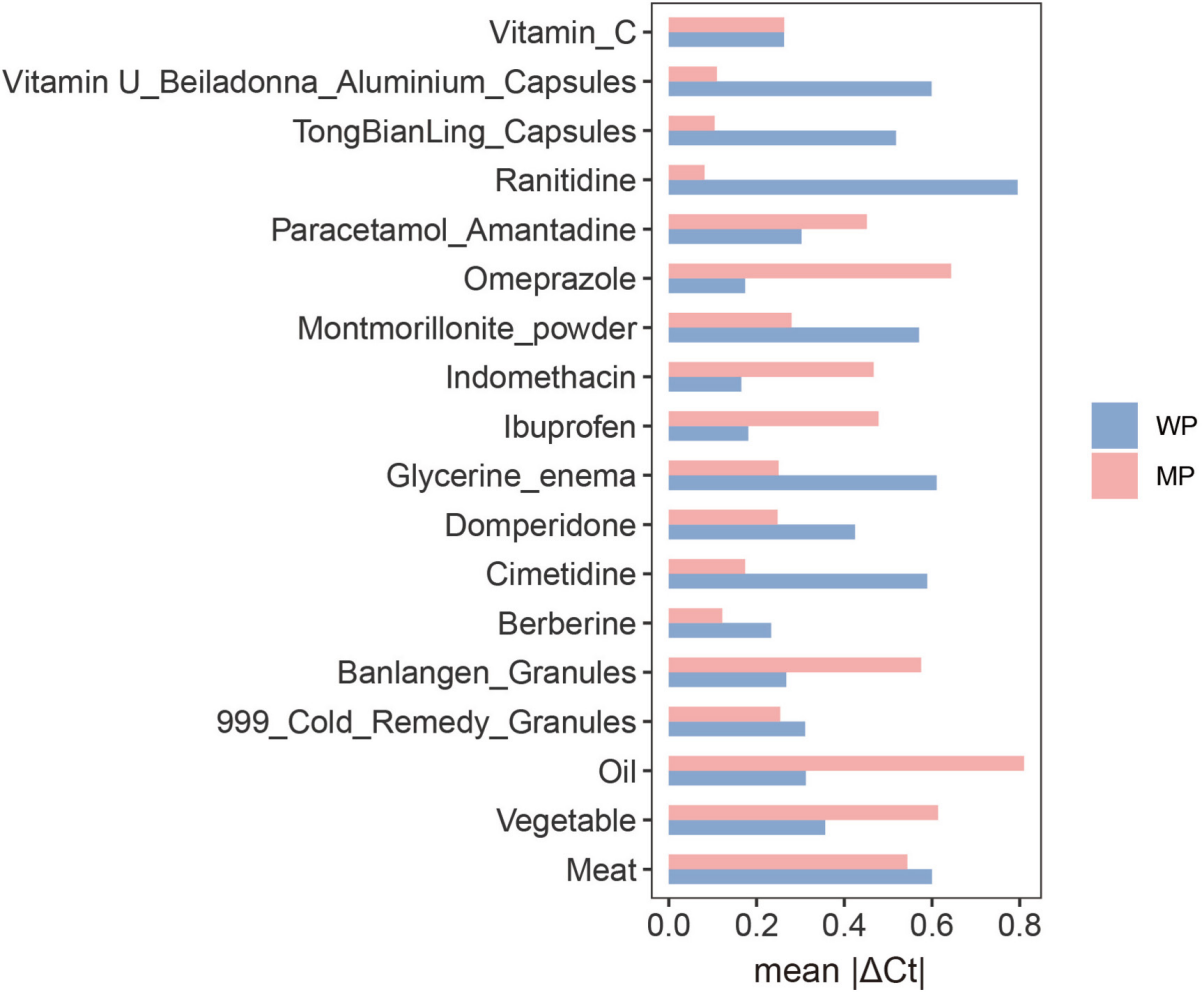
Effects of diet and 15 commonly used drugs for upper respiratory tract infection and gastrointestinal symptoms on fecal *m3* levels in vitro. |ΔCt| = Cttreated − Ctcontrol was used to describe the difference in fecal *m3* level between diet‐ or drug‐treated samples and controls. Mean |ΔCt| was the mean level of |ΔCt| from triplicated tests. WP means fecal *m3* weak‐positive. MP means fecal *m3* moderate‐positive.

**FIGURE 4:**
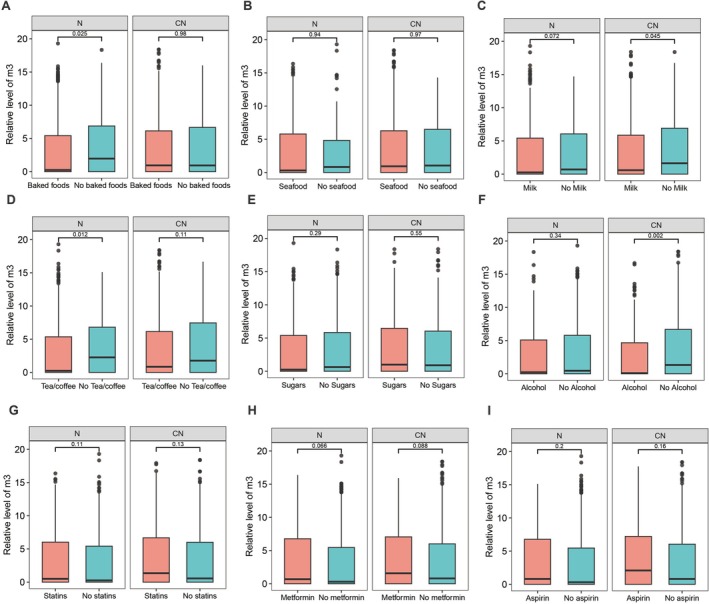
Effects of diet and drugs on fecal *m3* in Cohort I. Relative levels of fecal *m3* stratified by whether taking baked foods (A), seafood (B), milk or dairy products (C), tea or coffee (D), sugars (E), alcoholic drinks (F), statins (G), metformin (H), and aspirin (I) among normal controls (N) and subjects with colorectal neoplasia (CN, colorectal cancer or adenoma).

We tested the effects of three dietary components, including meat, vegetables, and oil, on fecal *m3* levels in vitro. All the diet‐treated samples and negative controls from *m3*‐weak‐positive and *m3*‐moderate‐positive samples showed fecal *m3* positive. |ΔCt| in the *m3*‐weak‐positive group was < 1.5, and |ΔCt| in the *m3*‐moderate‐positive group was < 1.0 (Figure 3). All the diet‐treated samples and negative controls from *m3*‐negative samples showed *m3*‐negative. Next, we compared the relative abundance of fecal *m3* stratified by taking specific diets or not in subjects with colorectal neoplasia (CRC and adenomas) and normal controls. The dietary intake information was shown in Table 2. To avoid sample size bias, we only compared diets that were not taken by > 10% of subjects in each group (Figure 4A–F). Intake of baked foods, seafood, tea or coffee, or sugars did not show significant effects on the relative levels of fecal *m3* in subjects with colorectal neoplasia. Intake of milk or dairy products (*p* = 0.045) and alcoholic drinks (*p* = 0.002) was associated with lower levels of *m3* in subjects with colorectal neoplasia, but not in normal controls (Figure 4C,F).

**TABLE 2:** Dietary information of Cohort I.

Diet	All (*N* = 1765)	Normal (*N* = 801)	Colorectal neoplasia (*N* = 964)
Pasta, *n* (%)			
Yes	1667 (94.4)	763 (95.3)	904 (93.8)
No	98 (5.6)	38 (4.7)	60 (6.2)
Bread, *n* (%)			
Yes	1589 (90.0)	723 (90.3)	866 (89.8)
No	176 (10.0)	78 (9.7)	98 (10.2)
Baked foods, *n* (%)			
Yes	1444 (81.8)	661 (82.5)	783 (81.2)
No	321 (18.2)	140 (17.5)	181 (18.8)
Meats, *n* (%)			
Yes	1736 (98.4)	787 (98.3)	949 (98.4)
No	29 (1.6)	14 (1.7)	15 (1.6)
Seafood, *n* (%)			
Yes	1549 (87.8)	694 (86.6)	855 (88.7)
No	216 (12.2)	107 (13.4)	109 (11.3)
Vegetables, *n* (%)			
Yes	1752 (99.3)	795 (99.3)	957 (99.3)
No	13 (0.7)	6 (0.7)	7 (0.7)
Fruits, *n* (%)			
Yes	1699 (96.3)	775 (96.8)	924 (95.9)
No	66 (3.7)	26 (3.2)	40 (4.1)
Milk and dairy products, *n* (%)			
Yes	1194 (67.6)	556 (69.4)	638 (66.2)
No	571 (32.4)	245 (30.6)	326 (33.8)
Soup, *n* (%)			
Yes	1642 (93.0)	750 (93.6)	892 (92.5)
No	123 (7.0)	51 (6.4)	72 (7.5)
Tea or coffee, *n* (%)			
Yes	1499 (84.9)	655 (81.8)	844 (87.6)
No	266 (15.1)	146 (18.2)	120 (12.4)
Sugars, *n* (%)			
Yes	1006 (57.0)	454 (56.7)	552 (57.3)
No	759 (43.0)	347 (43.3)	412 (42.7)
Alcoholic drinks, *n* (%)			
Yes	366 (20.7)	161 (20.1)	205 (21.3)
No	1399 (79.3)	640 (79.9)	759 (78.7)
Vitamin pills, *n* (%)			
Yes	534 (30.3)	276 (34.5)	258 (26.8)
No	1231 (69.7)	525 (65.5)	706 (73.2)

*Note:* Colorectal neoplasia refers to colorectal cancer and adenoma.

### 3.4 Impact of Drugs on Fecal *m3*

We also tested the effects of 15 commonly used drugs for upper respiratory tract infection and gastrointestinal symptoms on fecal *m3* levels in vitro. These drugs included non‐steroidal anti‐inflammatory drugs, acetaminophen, vitamins, antacids, and proton pump inhibitors. All the drug‐treated samples and negative controls from *m3*‐weak‐positive and *m3*‐moderate‐positive samples showed positivity for fecal *m3*. |ΔCt| in the *m3*‐weak‐positive group was < 1.5, and |ΔCt| in the *m3*‐moderate‐positive group was < 1.0 (Figure 3). All the drug‐treated samples and negative controls from *m3*‐negative samples showed negativity for *m3*. Collectively, these in vitro data showed that fecal *m3* was not affected by drugs. Next, we assessed the effect of three types of drugs for chronic diseases, including statins, metformin and aspirin, on fecal *m3*. We determined if there were any differences in fecal *m3* levels in colorectal neoplasia in subjects who were on certain drugs (Figure 4G–I). The relative level of *m3* in colorectal neoplasia was 1.35 (interquartile range, IQR 6.68) versus 0.56 (IQR 6.00) (*p* = 0.130) in subjects with and without statins, 1.58 (IQR 7.06) versus 0.81 (IQR 6.01) (*p* = 0.088) in subjects with and without metformin, and 2.09 (IQR 7.21) versus 0.81 (IQR 6.04) (*p* = 0.160) in subjects with and without aspirin. There was also no difference in *m3* levels in control subjects with and without these drugs (statin 0.50 (IQR 6.02) versus 0.26 (IQR 5.42), *p* = 0.110; metformin 0.69 (IQR 6.77) versus 0.30 (IQR 5.46), *p* = 0.068; aspirin 0.81 (IQR 6.78) versus 0.31 (IQR 5.46), *p* = 0.200). Overall, we found that statins, metformin, and aspirin did not show significant effects on fecal *m3* in subjects with adenoma, CRC, or normal controls.

### 3.5 Mixed Effect of Diet and Drugs on Fecal *m3* in Colorectal Neoplasia

To mimic the actual effects of diet and drugs on fecal *m3* in individuals with colorectal neoplasia, we used a logistic regression model to adjust clinical factors, including age, gender, smoking and drinking status, comorbidities (hypertension, diabetes mellitus, hyperlipidemia), and family history of CRC (Table 3). We used the median of the relative level of fecal *m3* as the cut‐off to further divide the colorectal neoplasia into high‐level and low‐level groups. In the multivariate analysis, which included variables with *p*‐value < 0.1 in the univariate analysis, apart from the presence of advanced neoplasia (Odd ratio, OR 1.35, 95% confidence interval, CI 1.02–1.81, *p* = 0.039), age > 65 years (OR 1.46, 95% CI 1.09–1.95, *p* = 0.011) was the only independent factor positively associated with high‐level fecal *m3* in subjects with colorectal neoplasia. After adjusting for clinical factors, fecal *m3* was not independently affected by diet and drugs.

**TABLE 3:** Impact of diet and drugs on fecal *m3* in colorectal neoplasia.

Variable	Univariate analysis		Multivariate analysis	
	OR (95% CI)	*p*	OR (95% CI)	*p*
Age > 65 years	1.58 (1.20–2.10)	0.001	1.46 (1.09–1.95)	0.011
Gender, male	0.89 (0.69–1.15)	0.364	—	—
BMI > 28	0.73 (0.52–1.03)	0.070	0.71 (0.50–1.00)	0.053
Smoking status				
Ex/non‐smoker	Ref.	—	—	—
Current smoker	1.22 (0.80–1.87)	0.350	—	—
Drinking status				
Ex/non‐drinker	Ref.	—	—	—
Current drinker	0.71 (0.47–1.06)	0.097	0.95 (0.55–1.64)	0.852
Comorbidities				
Hypertension	1.18 (0.91–1.53)	0.218	—	—
Diabetes mellitus	1.29 (0.99–1.69)	0.060	1.14 (0.85–1.53)	0.394
Hyperlipidemia	1.11 (0.86–1.44)	0.4419	—	—
Concurrent drugs				
Statins	1.32 (1.02–1.70)	0.033	1.15 (0.85–1.56)	0.350
Metformin	1.23 (0.89–1.69)	0.208	—	—
Aspirin	1.42 (0.99–2.02)	0.051	1.26 (0.86–1.56)	0.235
Family history of CRC	0.79 (0.55–1.14)	0.208	—	—
Diet intake				
Seafood	0.97 (0.65–1.44)	0.867	—	—
Milk or dairy products	0.76 (0.58–0.99)	0.042	0.77 (0.59–1.02)	0.069
Tea or coffee	1.32 (0.90–1.95)	0.158	—	—
Sugars	1.04 (0.81–1.335)	0.738	—	—
Alcoholic drinks	0.63 (0.46–0.85)	0.003	0.67 (0.44–1.03)	0.070
Vitamin pills	0.89 (0.67–1.18)	0.415	—	—
Presence of advanced neoplasia	1.35 (1.02–1.79)	0.035	1.35 (1.02–1.81)	0.039

*Note:* Advanced neoplasia included advanced adenoma and colorectal cancer.

Abbreviation: BMI, body mass index.

## 4 Discussion

The current study determined the characteristics of a novel bacterial gene marker *m3* in colorectal neoplasia, using two independent cohorts from Hong Kong and five cities in the Chinese mainland. The prevalence of fecal *m3* was higher in CRC (82.4%) than in adenomas (63.9%) or controls (63.4%) across different regions. This was, to our knowledge, the first and largest cohort to show this characteristic, which demonstrated the common presence of *m3* across different geographical regions. We also found significant differences in the fecal level of *m3* between AA versus non‐advanced adenoma (NAA) or controls, and ROC analysis indicated the diagnostic potential of fecal *m3* in AA with an AUROC of 0.604 (*p* < 0.001), echoing the potential of fecal *m3* as a non‐invasive biomarker for the diagnosis of AA, a clinically important precursor of CRC [16, 17].

Our dietary data showed that intake of milk and dairy products tended to be associated with lower levels of fecal *m3* in subjects with colorectal neoplasia, but not in normal controls. A recent systematic review showed that high consumption of total dairy products was associated with a consistent significant decrease in CRC risk [18]. Another cohort study also demonstrated that regular yogurt intake was associated with a 27% decrease in odds of colorectal adenoma among women [19]. However, the underlying mechanism of the protective effects of milk or dairy products in colorectal neoplasia remains unclear. Our study showed the negative association between intake of milk or dairy products and fecal *m3* in subjects with colorectal neoplasia, suggesting that milk or dairy products may reduce the risk of CRC or adenoma by inhibiting the bacterium *Lachnoclostridium*. Further mechanism studies are needed to validate our hypothesis.

Our in vitro experiment tested the effect of common diet components and commonly prescribed drugs on fecal *m3* and showed that fecal *m3* was not affected by these diets and drugs in vitro, which supported the real‐world application of fecal *m3* as a reliable and convenient non‐invasive diagnostic biomarker for colorectal neoplasia. Individuals can continue their usual diet and drugs when undergoing fecal *m3* tests, without interrupting their usual lifestyle.

Furthermore, we conducted a multivariate analysis using a logistic regression model to adjust for clinical factors and evaluate the impact of diet and drugs on fecal *m3*. This analysis aimed to assess the potential of fecal *m3* as a personalized diagnostic tool. Only age was the independent factor positively associated with high‐level fecal *m3*. Most importantly, fecal levels of *m3* were not affected by diet or common drugs for acute and chronic diseases. This result suggested the stability and reliability of fecal *m3*, highlighting its potential as a biomarker for colorectal neoplasia diagnosis or even CRC screening.

We found that 1.2% (*n* = 21) of subjects in Cohort I reported an antibiotic history within 30 days before fecal sample collection. To address the limitation of antibiotic exposure in these subjects and further minimize confounding bias, we conducted a post hoc propensity score‐matched subgroup analysis. Using the R MatchIt package with 1:1 matching based on age, gender, and disease, we included 42 subjects (i.e., 21 antibiotic‐exposed and 21 antibiotic‐naïve subjects) comprising 11 pairs of normal controls and 10 pairs of adenoma patients. Matching achieved a balance between antibiotic‐exposed and antibiotic‐naïve groups with comparable sex distribution (14 females, 7 males per group), mean age (64.0 ± 11.3 vs. 64.0 ± 11.4 years), and disease. Among 21 antibiotic‐exposed subjects, exposures included but were not limited to Augmentin, amoxycillin, clarithromycin and levofloxacin at doses ranging from 375 to 1000 mg, with a median of 6 days (range 0–26 days) between the last antibiotic dose and fecal sample collection. No significant difference was found in *m3* levels between antibiotic‐exposed and antibiotic‐naïve groups (*p* > 0.05), regardless of the presence of adenomas. In subjects with > 4 days between the last antibiotic dose and fecal sample collection, *m3* levels remained indistinguishable from antibiotic‐naïve individuals (*p* > 0.05). Days since last antibiotic dose, antibiotic class, or dosage did not significantly influence *m3* levels (*p* > 0.05). Taken together, these analyses demonstrated robust stability and reliability of *m3* as a fecal bacterial gene marker after antibiotic regimens.

There were several limitations in the current study. First, the current study only included a Chinese population, thus confirmation in cohorts from other ethnic backgrounds and with different dietary patterns will be important to establish generalizability. Second, dietary intake was assessed using a self‐administered qualitative food intake questionnaire, which is susceptible to recall bias. Apart from diet, exercise is also an important factor affecting gut microbiota. Future prospective studies incorporating more granular assessments of diet and physical activity could further clarify the effects of host lifestyle factors on the gut microbiome and fecal *m3* levels. Third, our in vitro experiments and clinical data covered a range of commonly used drugs, and the potential effects of less frequently used or untested agents cannot be fully excluded. Fourth, different DNA extraction kits and qPCR conditions were used across the two cohorts. Although internal cross‐validation suggested that their impact on *m3* measurements was likely minimal, standardized protocols would be warranted in future studies. The sample size in the in vitro experiment was relatively small, reflecting its pilot nature. A larger experimental series will be important for validation. Lastly, the small number of antibiotic‐exposed subjects (*n* = 21) limited statistical power, necessitating the propensity score matching approach to minimize confounding. While matching achieved an excellent balance on key covariates, larger prospective studies would provide more definitive evidence.

## 5 Conclusion

Fecal *m3* is a promising non‐invasive diagnostic marker for colorectal neoplasia diagnosis. The fecal level and diagnostic performance of this bacterial gene marker were not affected by diet or common drugs, suggesting its reliability for colorectal neoplasia detection.

## Ethics Statement

This study was approved by the Joint Chinese University of Hong Kong–New Territories East Cluster Clinical Research Ethics Committee (CREC reference no. 2022.170) and the Ethics Committee of Beijing Friendship Hospital, Capital Medical University (No. 2022‐P2‐084).

## Consent

Informed consent was obtained from all participants prior to inclusion.

## Supporting information


**Figure S1:** Prevalences (A) and relative levels (B) of fecal *m3* in the combined cohort (Cohort I and II) among normal controls (N), non‐advanced adenoma (NAA), advanced adenoma (AA), and colorectal cancer (CRC) groups. Relative levels of fecal *m3* in the separated Cohort I (C) and Cohort II (D) among normal controls, NAA, AA, and CRC groups. Prevalences (E) and relative levels (F) of fecal *m3* in the Cohort II stratified by TNM stage of colorectal cancer. *, *p* < 0.05; **, *p* < 0.01; ***, *p* < 0.001, ****, *p* < 0.0001.

**Data S1:** Supporting information.

**Data S2:** Supporting information.

**Table S1:** Final concentration of diet and drug in vitro experiment.
**Table S2:** Clinical characteristics of Cohort II.

## Data Availability

De‐identified individual data from this article will be made available to the corresponding author on reasonable request.
