# Supplementary figures and images for: Impact of Diet and Drugs on Fecal Lachnoclostridium Gene Marker (m3) in Non‐Invasive Diagnosis of Colorectal Neoplasia
Source: J Gastroenterol Hepatol. 2026 Feb 13;41(4):1213–22. doi: 10.1111/jgh.70295 (PMC13058778; doi:10.1111/jgh.70295)

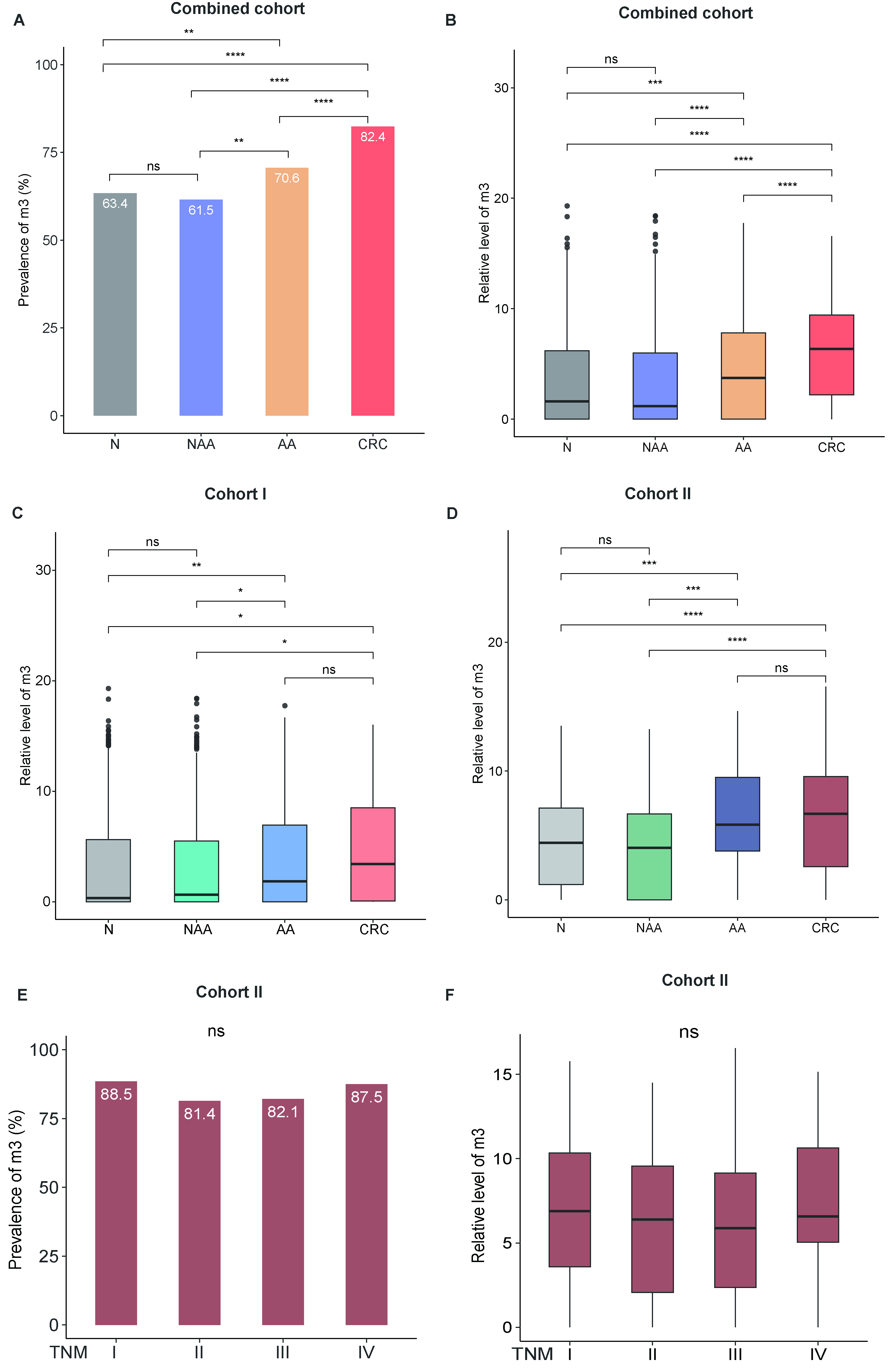

Supplement: Supplementary file 1 — Figure S1: Prevalences (A) and relative levels (B) of fecal m3 in the combined cohort (Cohort I and II) among normal controls (N), non‐advanced adenoma (NAA), advanced adenoma (AA), and colorectal cancer (CRC) groups. Relative levels of fecal m3 in the separated Cohort I (C) and Cohort II (D) among normal controls, NAA, AA, and CRC groups. Prevalences (E) and relative levels (F) of fecal m3 in the Cohort II stratified by TNM stage of colorectal cancer. *, p < 0.05; **, p < 0.01; ***, p < 0.001, ****, p < 0.0001. [file JGH-41-1213-s001.tif]
