# Supplementary material for: Impact of Diet and Drugs on Fecal Lachnoclostridium Gene Marker (m3) in Non‐Invasive Diagnosis of Colorectal Neoplasia
Source: J Gastroenterol Hepatol. 2026 Feb 13;41(4):1213–22. doi: 10.1111/jgh.70295 (PMC13058778; doi:10.1111/jgh.70295)
Supplement: Supplementary file 2 — Data S1: Supporting information. [file JGH-41-1213-s004.docx]

**Dietary questionnaire**

Did you have the following food or drinks last 2 weeks?

|  | Yes | No |
| --- | --- | --- |
| 1. Pasta, pasta salad, or noodles (e.g. spaghetti, ravioli, pasta salad) | □_1_ | □_2_ |
| 1. Bread (e.g. sliced bread) | □_1_ | □_2_ |
| 1. Sweet baked foods (e.g. cakes, muffin, muesli bars) | □_1_ | □_2_ |
| 1. Meats (e.g. pork, beef, lamb, luncheon meat, pork ribs, chicken) | □_1_ | □_2_ |
| 1. Seafood (e.g. clams, salmon, fish fingers, crab cakes) | □_1_ | □_2_ |
| 1. Vegetables | □_1_ | □_2_ |
| 1. Fruits and products | □_1_ | □_2_ |
| 1. Milk and dairy products (e.g. fresh milk, milk powder, yogurt, ice cream) | □_1_ | □_2_ |
| 1. Soup | □_1_ | □_2_ |
| 1. Tea and coffee | □_1_ | □_2_ |
| 1. Sugars and syrups (e.g. cane sugar, white sugar, syrup) | □_1_ | □_2_ |
| 1. Alcoholic drinks (e.g. beer, wine, cider, spirits) | □_1_ | □_2_ |
| 1. Vitamin pills (e.g. fish oil capsules, multivitamin pills) | □_1_ | □_2_ |
| 1. Others (Please specify: _____________________________________________________) | | |
| 1. Others (Please specify: _____________________________________________________) | | |
| 1. Others (Please specify: _____________________________________________________) | | |

Subject’s Signature: _______________________ Date:__ __ / __ __ / 20 __ __

Interviewer’s Name: _______________ Signature: ____________ Date:__ __ / __ __ / 20 __ __
