# Supplementary material for: Impact of Diet and Drugs on Fecal Lachnoclostridium Gene Marker (m3) in Non‐Invasive Diagnosis of Colorectal Neoplasia
Source: J Gastroenterol Hepatol. 2026 Feb 13;41(4):1213–22. doi: 10.1111/jgh.70295 (PMC13058778; doi:10.1111/jgh.70295)
Supplement: Supplementary file 3 — Data S2: Supporting information. [file JGH-41-1213-s003.docx]

1. **Effects of different qPCR cycle numbers for bacterial gene marker *m3***

**Protocol**

Target bacterial *m3* plasmid and 6 non-target bacterial DNA samples were tested by quantitative PCR (qPCR) (**Table 1**). Other qPCR conditions were kept constant while three different cycle numbers (35, 40, and 45) were assessed. This experiment aimed to compare the effects of different cycle numbers on the amplification curves of the target bacterial DNA and assess any non-specific amplification of non-target bacterial DNA.

**Table 1: Samples for cycle number optimization experiment**

| **Category** | **Bacterial DNA sample** |
| --- | --- |
| Target bacterial marker | *Lachnoclostridium* (*m3*) |
| Non-target bacterial marker | *Fusobacterium nucleatum subspecies* (*Fn28*) |
|  | *Bacteroides thetaiotaomicron* (*Bt32*) |
|  | *Hungatella xylanolytica* (*Hx08*) |
|  | *Christensenella minuta* (*Cm07*) |
|  | *Bacteroides fragilis* (*Bf09*) |
|  | *Escherichia coli* (*Ec73*) |

**Results**

**Amplification curves**

The amplification curves of different bacterial liquid DNAs at various cycle numbers were shown in **Figure 1**. For the target bacterial marker (*m3*), both the FAM channel and the VIC channel exhibited typical “S”-shaped amplification curves. For the non-target bacterial marker, the VIC channel showed a typical “S”-shaped amplification curve, while the FAM channel showed no non-specific amplification, which met the requirements.

When the cycle number reached 35, the amplification for the target bacterial DNA reached a plateau for both the FAM and VIC channels. The amount of amplification products tended to stabilize as the cycle number increased. For non-target bacterial DNA, even when the cycle number gradually increased to 45, there was still no non-specific amplification in the FAM channel, indicating that increasing the cycle number did not lead to non-specific amplification. Therefore, based on the amplification curves, cycle numbers between 35 and 45 were considered acceptable.


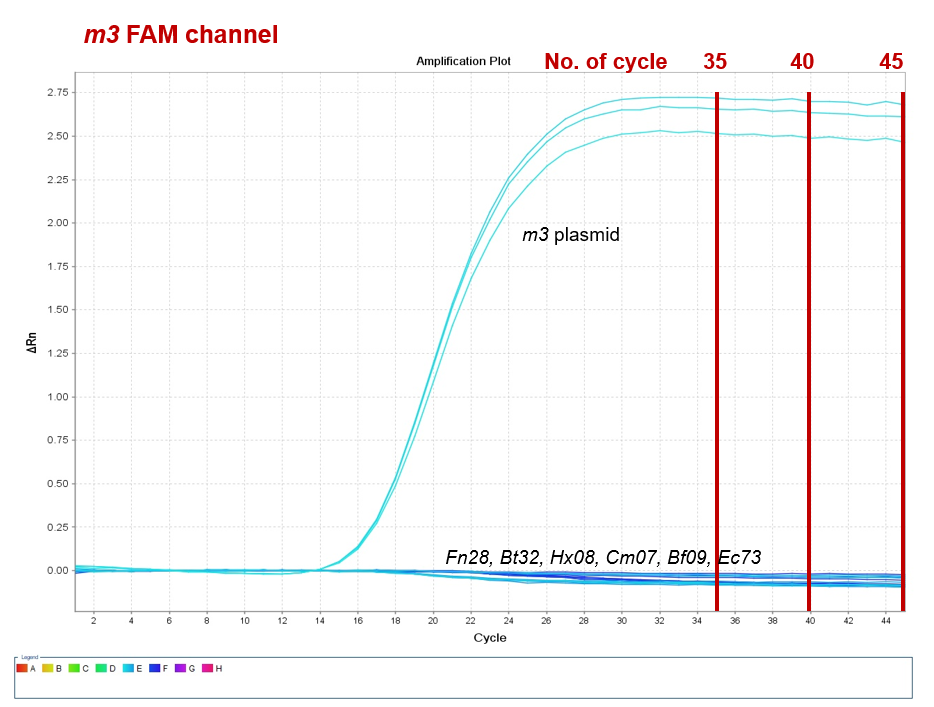


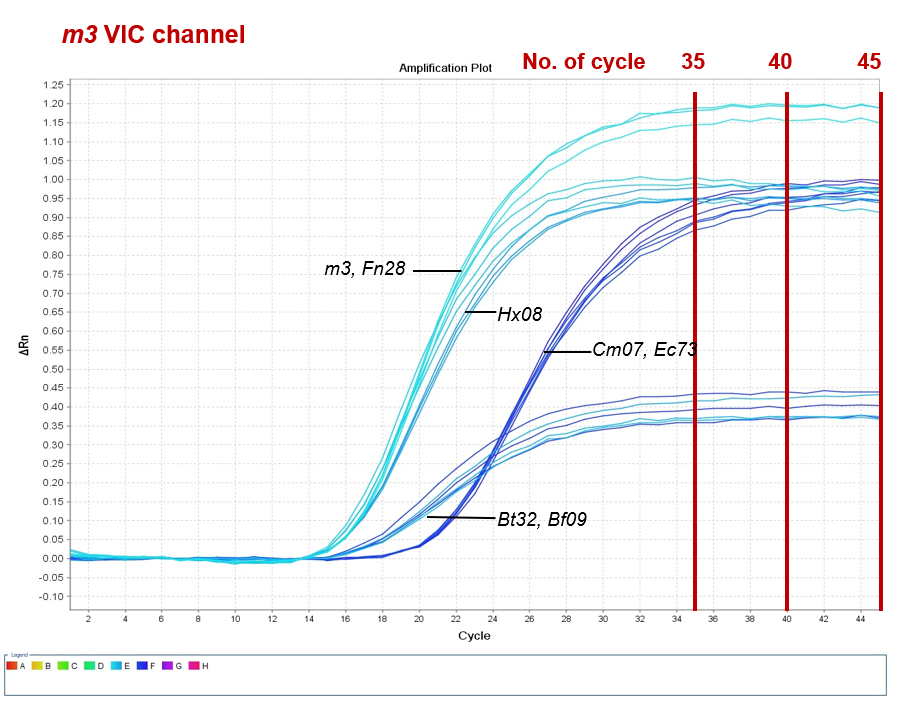


**Figure 1: Detection results at different cycle numbers**

**Ct values**

The results of Ct values for bacterial DNA at different qPCR cycle numbers were shown in **Table 2**. When the cycle number reached 35, amplification had reached a plateau. The Ct values remained consistent across different cycle numbers, and no non-specific amplification appeared for non-target bacteria. Thus, based on the Ct values of bacterial DNA amplification, cycle numbers from 35 to 45 were considered acceptable.

**Table 2: Average Ct values for bacterial DNA at different cycle numbers**

| **Bacterial DNA** | **Channel** | **Ct values of different cycle numbers** | | |
| --- | --- | --- | --- | --- |
|  |  | **35** | **40** | **45** |
| *m3* | FAM | 15.33 | 15.33 | 15.33 |
|  | VIC | 16.23 | 16.23 | 16.23 |
| *Fn28* | FAM | Unde | Unde | Unde |
|  | VIC | 15.86 | 15.86 | 15.86 |
| *Bt32* | FAM | Unde | Unde | Unde |
|  | VIC | 18.75 | 18.75 | 18.75 |
| *Hx08* | FAM | Unde | Unde | Unde |
|  | VIC | 16.26 | 16.26 | 16.26 |
| *Cm07* | FAM | Unde | Unde | Unde |
|  | VIC | 20.92 | 20.92 | 20.92 |
| *Bf09* | FAM | Unde | Unde | Unde |
|  | VIC | 18.47 | 18.47 | 18.47 |
| *Ec73* | FAM | Unde | Unde | Unde |
|  | VIC | 21.07 | 21.07 | 21.07 |

In conclusion, the amplification results of bacterial liquid DNA indicated that cycle numbers from 35 to 45 did not affect the qPCR product yield of the target bacteria, and there was no non-specific amplification for non-target bacteria. Therefore, cycle numbers can be set to 35-45.

1. **Determination of the range of |ΔCt| threshold**

Two selected *m3-*moderate-positive *and m3-*week-positive samples were repeated qPCR test 20 times. Ct values were listed in Table 3. The ranges of the Ct value for *m3*-moderate-positive and *m3*-week-positive samples were 0.63 and 1.19, respectively.

According to this result, we set |ΔCt|<1.0 and |ΔCt|<1.5 for *m3*-moderate-positive and *m3*-week-positive samples, respectively.

**Table 3. Ct values and range of *m3*-moderate-positive and *m3*-week-positive samples**

| ***m3*-moderate-positive sample** | | | ***m3*-weak-positive sample** | | |
| --- | --- | --- | --- | --- | --- |
| **Sample ID** | **No.** | **Ct value** | **Sample ID** | **No.** | **Ct value** |
| Y2-376 | 1 | 30.85 | GZ-114 | 1 | 33.95 |
|  | 2 | 30.69 |  | 2 | 34.35 |
|  | 3 | 31.00 |  | 3 | 33.96 |
|  | 4 | 31.22 |  | 4 | 33.99 |
|  | 5 | 30.99 |  | 5 | 34.61 |
|  | 6 | 31.26 |  | 6 | 33.67 |
|  | 7 | 31.21 |  | 7 | 34.76 |
|  | 8 | 31.08 |  | 8 | 34.13 |
|  | 9 | 30.63 |  | 9 | 34.01 |
|  | 10 | 31.04 |  | 10 | 34.75 |
|  | 11 | 30.81 |  | 11 | 34.29 |
|  | 12 | 30.99 |  | 12 | 33.58 |
|  | 13 | 30.96 |  | 13 | 34.06 |
|  | 14 | 30.91 |  | 14 | 33.98 |
|  | 15 | 30.91 |  | 15 | 34.64 |
|  | 16 | 31.12 |  | 16 | 34.73 |
|  | 17 | 30.87 |  | 17 | 33.59 |
|  | 18 | 30.86 |  | 18 | 34.69 |
|  | 19 | 30.98 |  | 19 | 33.66 |
|  | 20 | 30.76 |  | 20 | 33.96 |
| **Mean** | / | 30.96 |  | / | 34.17 |
| **Coefficient of variation** | / | 0.55% |  | / | 1.19% |
| **Range** | / | **0.63** |  | / | **1.19** |
