# Supplementary material for: Impact of Diet and Drugs on Fecal Lachnoclostridium Gene Marker (m3) in Non‐Invasive Diagnosis of Colorectal Neoplasia
Source: J Gastroenterol Hepatol. 2026 Feb 13;41(4):1213–22. doi: 10.1111/jgh.70295 (PMC13058778; doi:10.1111/jgh.70295)
Supplement: Supplementary file 4 — Table S1: Final concentration of diet and drug in vitro experiment. Table S2: Clinical characteristics of Cohort II. [file JGH-41-1213-s002.docx]

**Supplementary Table 1. Final concentration of diet and drug *in vitro* experiment**

| **Name** | **Final concentration** |
| --- | --- |
| Vitamin U, Belladonna Aluminum Capsules | 6.84mg/mL |
| Domperidone | 0.14mg/mL |
| Ranitidine | 3 mg/mL |
| Cimetidine | 2mg/mL |
| Berberine | 6 mg/mL |
| Montmorillonite powder | 12 mg/mL |
| TongBianLing Capsules | 7.25mg/mL |
| Glycerin enema | 0.83 mg/mL |
| Vitamin C | 14μg/mL |
| 999 Cold Remedy Granules | 7.5 mg/mL |
| Paracetamol Amantadine | 0.245 mg/mL |
| Ibuprofen | 1.07 mg/mL |
| Banlangen Granules | 10 mg/mL |
| Omeprazole | 2.82 mg/mL |
| Indomethacin | 0.75 mg/mL |
| Meat | 10 mg/mL |
| Vegetable | 10 mg/mL |
| Oil | 300mg/mL |

**Supplementary Table 2. Clinical characteristics of Cohort II**

| **Variable** | **Normal (N = 313)** | **Adenoma (N = 139)** | **CRC (N = 285)** |
| --- | --- | --- | --- |
| Age, mean (SD), year | 55.0 (8.9) | 59.1 (9.1) | 61.2 (8.3) |
| Gender, male (%) | 122 (39.0) | 121 (60.5) | 123 (59.4) |
| City, n (%) |  |  |  |
| Beijing | 61 (19.5) | 88 (44.0) | 64 (22.5) |
| Shanghai | 58 (18.5) | 38 (19.0) | 60 (21.1) |
| Guangzhou | 39 (12.5) | 10 (5.0) | 7 (2.5) |
| Kunming | 147 (47.0) | 60 (30.0) | 98 (34.4) |
| Xi’an | 8 (2.6) | 4 (2.0) | 56 (19.6) |
| TNM stage, n (%) |  |  |  |
| I | - | - | 26 (9.1) |
| II | - | - | 86 (30.2) |
| III | - | - | 95 (33.3) |
| IV | - | - | 24 (8.4) |

CRC, colorectal cancer.
